# Acknowledgement to Reviewers of *Geriatrics* in 2019

**DOI:** 10.3390/geriatrics5010003

**Published:** 2020-01-20

**Authors:** Geriatrics Editorial Office

**Affiliations:** MDPI, St. Alban-Anlage 66, 4052 Basel, Switzerland

The editorial team greatly appreciates the reviewers who have dedicated their considerable time and expertise to the journal’s rigorous editorial process over the past 12 months, regardless of whether the papers are finally published or not. In 2019, a total of 60 papers were published in the journal, with a median time to first decision of 20 days and a median time from submission to publication of 47 days. The editors would like to express their sincere gratitude to the following reviewers for their generous contribution in 2019:
Affoo, RebeccaMatta, JoaneAllen, Jacqueline E.Mazzola, PaoloAmini, RezaMcCulloch, JoeApfeldorf, William JayMelgaard, DorteArthur, Susan T.Menahem, SolomonAspinall, PeterMeyssonnier, VaninaAssari, ShervinMichels, Alexander J.Baeyens, Jean-PierreMiddleton, MarkBanerjee, SudipMihaicuta, StefanBarczi, StevenMilazzo, MarioBarrera, Marissa A.Miles, AnnaBartfay, EmmaMistiaen, WilhelmBeghi, EttoreMohamed, MostafaBerkowsky, Ronald W.Mok, Daniel Kam-WahBerry, TriciaMold, Matthew J.Bohannon, Richard W.Mooijaart, SimonBolinger, ChristopherMorandi, AlessandroBotturi, AndreaMorey, Miriam C.Brooke, JoanneMugueta-Aguinaga, IranzuBujnowska-Fedak, Maria MagdalenaMukaetova-Ladinska, Elizabeta BByeon, HaewonMulheren, RachelCassarino, MaricaMurray-Smith, AmberCauli, OmarNaruishi, KojiCerejeira, JoaquimNashef, LinaChiu, Yu-Lung MarcusNeedell, Nancy J.Choi, Shing WanNigwekar, Sagar U.Cohen, AliciaNilsson, AndreasColgrove, YvonneNorman, Gregory J.Corica, FrancescoOrtega, OmarCosta Ramos, DanielPachana, Nancy A.Cravello, LucaPatel, Harnish P.Damsgaard, Else MariePecor, KeithDavies, Christina R.Petzold, GuillermoDe Berardis, DomenicoPijpers, RoosDeCherrie, LindaPilling, Luke C.Di Lorenzo, FrancescoPitts, TeresaDickerson, AnnePizzorni, NicoleDong, Huan-JiPownall, SusanEcarnot, FionaPownall, SueEiroa-Orosa, Francisco JosePulle, Ranjeev ChrysanthEscher, AnneQuan, Stuart F.Fappi, AlanRaposio, EdoardoFernando, BinoshaReynolds, LoriFiest, Kirsten M.Rodrigues, Célia F.Franzen, Aaron B.Rogus-Pulia, NicoleFritz, ZoëRoss, SamuelFujimoto, YuhiSaito, SatoshiGambert, Steven R.Sakai, KotomiGeorge, StaceySantric Milicevic, Milena MGijón-Nogueron, GabrielSasegbon, AyodeleGodycki-Ćwirko, MaciekSaul, DominikGolden, RobynSawhney, Mona K.Goldzweig, GilSchmidt, HeikeGómez-López, PedroSchubert, Cathy C.Gu, YuchaoSeaman, AaronHaboubi, NadimSerrat, RodrigoHan, Der-ShengSganga, FedericaHauser, W. AllenShewmaker, Frank P.Hill, CameronShune, SamanthaHöglund, Anna T.Siedlecki, KarenHunt, Linda A.Silva, SamuelHuseth-Zosel, Andrea L.Singh, InderpalIbrahim, KindaSkingley, AnnIlluminati, GiulioSmith, Dylan M.Isaacson, MichalSmith, TobyIsoherranen, KirsiStarčević, DamirIyer, ChitraStewart Williams, JenniferJain, Rajesh KSwaine, Ian L.Jancke, LutzTakura, TomoyukiJansen, PetraTao, DaJones, Corinne A.Tarazona-Santabalbina, Francisco JoséKang, Tong HoThiamwong, LaddaKatlic, Mark R.Thomas, ChristineKeevil, Victoria L.Tomita, Machiko R.Keller, SusanaTzallas, AlexandrosKellner, Christopher P.Vaismoradi, MojtabaKenji, Sakaivan Hoof, JoostKirshner, HowardVan Rijn, MarjonKrishna, Somashekar G.Velusamy, VijayalakshmiKunutsor, Setor K.Vincensi, BarbaraLecky, Donna MVose, Alicia K.Lee, Yu-ChenWadhwaniya, ShirinLeppik, Ilo E.Walters, MatthewLeto, TheresaWatanabe, TomonoriLindroth, HeidiWatson, RogerLiu, FangWatts, StephanieLiu, Li-FanWieland, G. DarrylLojo-Seoane, CristinaWion, Rachel K.Macijauskienė, JūratėWitt, MartinMadhavan, AarthiWukich, DaneMagriplis, EmmanuelaYang, Wei-HsunMajeed, HaroonYu, TsungManninger, MartinZanini, MilkoMarcusson, JanZimmerli, WernerMarin, ConcepcióZuelsdorff, Megan L.

